# The Metabolic Syndrome and the immediate antihypertensive effects of aerobic exercise: a randomized control design

**DOI:** 10.1186/1471-2261-8-12

**Published:** 2008-06-10

**Authors:** Linda S Pescatello, Bruce E Blanchard, Jaci L Van Heest, Carl M Maresh, Heather Gordish-Dressman, Paul D Thompson

**Affiliations:** 1 Department of Kinesiology, University of CT, Storrs, CT, USA; 2 Department of Pathology, Hartford Hospital, Hartford, CT, USA; 3 Research Center for Genetic Medicine, Children's National Medical Center, Washington, DC, USA; 4 Division of Cardiology, Hartford Hospital, Hartford, CT, USA

## Abstract

**Background:**

The metabolic syndrome (Msyn) affects about 40% of those with hypertension. The Msyn and hypertension have a common pathophysiology. Exercise is recommended for their treatment, prevention and control. The influence of the Msyn on the antihypertensive effects of aerobic exercise is not known. We examined the influence of the Msyn on the blood pressure (BP) response following low (LIGHT, 40% peak oxygen consumption, VO_2_peak) and moderate (MODERATE, 60% VO_2_peak) intensity, aerobic exercise.

**Methods:**

Subjects were 46 men (44.3 ± 1.3 yr) with pre- to Stage 1 hypertension (145.5 ± 1.6/86.3 ± 1.2 mmHg) and borderline dyslipidemia. Men with Msyn (n = 18) had higher fasting insulin, triglycerides and homeostasis model assessment (HOMA) and lower high density lipoprotein than men without Msyn (n = 28) (p < 0.01). Subjects consumed a standard meal and 2 hr later completed one of three randomized experiments separated by 48 hr. The experiments were a non-exercise control session of seated rest and two cycle bouts (LIGHT and MODERATE). BP, insulin and glucose were measured before, during and after the 40 min experiments. Subjects left the laboratory wearing an ambulatory BP monitor for the remainder of the day. Repeated measure ANCOVA tested if BP, insulin and glucose differed over time among experiments in men without and with the Msyn with HOMA as a covariate. Multivariable regression analyses examined associations among BP, insulin, glucose and the Msyn.

**Results:**

Systolic BP (SBP) was reduced 8 mmHg (p < 0.05) and diastolic BP (DBP) 5 mmHg (p = 0.052) after LIGHT compared to non-exercise control over 9 hr among men without versus with Msyn. BP was not different after MODERATE versus non-exercise control between Msyn groups (p ≥ 0.05). The factors accounting for 17% of the SBP response after LIGHT were baseline SBP (β = -0.351, r^2 ^= 0.123, p = 0.020), Msyn (β = 0.277, r^2 ^= 0.077, p = 0.069), and HOMA (β = -0.124, r^2 ^= 0.015, p = 0.424). Msyn (r^2 ^= 0.096, p = 0.036) was the only significant correlate of the DBP response after LIGHT.

**Conclusion:**

Men without the Msyn respond more favorably to the antihypertensive effects of lower intensity, aerobic exercise than men with the Msyn. If future work confirms our findings, important new knowledge will be gained for the personalization of exercise prescriptions among those with hypertension and the Msyn.

## Background

Essential hypertension is often associated with a clustering of multiple risk factors for cardiovascular disease (CVD) and diabetes mellitus including abdominal adiposity, insulin resistance, impaired glucose tolerance, and dyslipidemia in addition to elevated blood pressure (BP) [1,2]. This clustering of multiple risk factors is referred to as the metabolic syndrome (Msyn) and affects about 30% of the general population [3] and 40% of those with hypertension [4]. The public health burden of hypertension [5] and the Msyn [4,6] continues to increase as the population ages and grows in size.

Participation in habitual physical activity and higher cardiovascular fitness are inversely related to the prevalence of hypertension [7] and the Msyn [8]. Lifestyle modifications are recommended for the prevention, treatment and control of these conditions, with exercise being a key component [7,9,10]. Some of the health benefit ascribed to aerobic exercise training programs is attributed to the acute or immediate effects that result from a single exercise session, effects that persist from one to three days depending on the CVD risk factor targeted [7,11]. The immediate antihypertensive effects of aerobic exercise, i.e., *postexercise hypotension *(PEH), result in BP decreases of 5–7 mm Hg that endure for up to 22 hr after the exercise session [7,12].

Insulin resistance and hyperinsulinemia are thought to be primary etiologic agents of hypertension and the Msyn [13-17]. Multiple mechanisms have been postulated to explain these associations including activation of the sympathetic nervous system, proliferation of vascular smooth muscle, altered cation transport, and increased sodium reabsorption. These derangements increase peripheral vasculature resistance, impair glucose tolerance, provoke dylipidemia and endothelial dysfunction, and elevate BP [18,15-19]. Mancia and colleagues [6] longitudinally assessed the relationship between the Msyn and early death among 2,013 adults from 25–74 yr. The associations they found among the Msyn and death from CVD and all causes were primarily explained by hypertension and hyperglycemia. Despite a common underlying pathophysiology and the importance of exercise in the treatment, prevention and control of hypertension and the Msyn, the influence of the Msyn on the immediate antihypertensive effects of aerobic exercise among those high BP is not known.

The primary purpose of this investigation was to examine the influence of the Msyn on the BP response following a session of low (LIGHT, 40% peak oxygen consumption, VO_2_peak) and moderate (MODERATE, 60% VO_2_peak) intensity, aerobic exercise among men with pre- to Stage 1 hypertension. Middle-aged adults with high BP and the Msyn are at higher CVD risk than those with high BP and without the Msyn [1,6,20]. Our work indicates that those more biologically predisposed to CVD, experience greater BP reductions immediately following a session of aerobic exercise than those less predisposed to CVD [21,22]. For these reasons, we hypothesized that men with pre- to Stage 1 hypertension and the Msyn would manifest PEH to a greater degree than men without the Msyn. Contrary to our hypothesis, men with high BP and without the Msyn responded more favorably to the immediate antihypertensive effects of lower intensity, aerobic exercise than men with high BP and the Msyn.

## Methods

### Subjects

Study volunteers were 46 men with pre- to Stage 1 hypertension [systolic BP (SBP) ≥ 130 and < 160 and/or diastolic BP (DBP) ≥ 85 and < 100 mm Hg]. Based upon the National Cholesterol Education Program criteria [2], 39.1% of these men presented with the Msyn. Volunteers were physically inactive, had no physical limitations that would prevent exercise, and did not smoke. Subjects were excluded if they had SBP ≥ 160 mm Hg and/or DBP ≥ 100 mm Hg, symptomatic atherosclerotic CVD, diabetes mellitus, asthma, thyroid dysfunction, pancreatitis, acute illness and/or were on antidepressant medication. Subjects signed an informed consent approved by the Institutional Review Boards of the University of Connecticut and Hartford Hospital.

Medications potentially influencing BP were stopped a minimum of 4 wk prior to testing. These medications included antihypertensives, antilipemic agents, aspirin, nonsteroidal anti-inflammatory agents, nutritional supplements except for a 1-a-day vitamin, cold medications, and herbal supplements. During the antihypertensive medication washout period, investigators regularly monitored BP. If the withdrawal of antihypertensive medication resulted in resting SBP ≥ 160 and/or DBP ≥ 100 mm Hg, men were excluded from further participation and resumed their medication regimen.

### Procedures

The procedures have been published in part previously [21-24]. Briefly, subjects participated in an orientation session to familiarize them with the study and ensure that their BP met the study criteria. During the orientation session, participants were instructed to maintain their normal diet throughout the study with the exception of a standard pre-experiment meal. The pre-experiment meal was consumed 2 hr prior to testing and consisted of 240 ml (1 cup) of low fiber cereal or a choice of one of the following: two slices of white toast, an English muffin, or a 8.9 cm (3.5 in) diameter plain bagel. This meal also included 120 ml (4 oz) of 2%, 1% or skim milk, and 240 ml (8 oz) of orange juice. Participants were also asked to refrain from any caffeinated beverage the morning of the testing sessions, and to only drink caffeinated [≤ 480 ml (2 cups)] and alcoholic (≤ 2 drinks/d) beverages in moderation throughout the study. Weight maintenance was defined as ± 2.25 kg (5.0 lb) of orientation weight and used as an indication the men were adhering to their normal dietary patterns during the study. Body weights were obtained prior to the graded cardiopulmonary exercise test and each of the three experiments to ensure weight maintenance.

The orientation session ended with attachment of an ambulatory BP monitor (Accutracker II automatic noninvasive ambulatory BP monitor, Suntech Medical Instruments Inc., Raleigh, NC) to each subject. The purpose of this ambulatory BP monitor session was to familiarize subjects with the unit and ensure their BP met the study inclusion criteria. Each time the same investigator attached the ambulatory BP monitor to a subject, a calibration check was done with a mercury sphygmomanometer using a t-tubule as per the manufacture's guidelines. The calibration check consisted of three test runs to ensure three successive ambulatory BP measurements were within 5 mm Hg of ascultatory BP measurements. The intra-investigator coefficient of variation between the ambulatory and ascultatory BP measurements made during these test runs was 0.7% for SBP and 1.8% for DBP. The monitor was programmed to record BP and heart rate (HR) approximately every 20 min. BP and HR were taken 3 times an hour until 2300 hr. Subjects left the laboratory with instructions to proceed with their typical activities, not to participate in formal exercise for the remainder of the day, to leave their arm still when the monitor was recording, and to return the monitor the following day. Upon returning the ambulatory BP monitor, a 12 hr fasting venous blood sample was obtained for determination of insulin, glucose and blood lipid-lipoproteins.

Participants then completed a maximum graded cardiopulmonary exercise stress test used to determine the exercise session workloads. The exercise test was done on a cycle ergometer (Monark Ergomedic 818E, Stockholm, Sweden) and consisted of continuous cycling at a cadence of 60 rev·min^-1 ^with the resistance increased 0.5 kp (30 W) every 2 min until volitional exhaustion. VO_2_peak was determined using breath by breath analysis of expired gases (Sensormedics Vmax 29 Metabolic Chart, SensorMedics Corp., Yorba Linda, CA). HR was recorded continuously with a 12 lead electrocardiogram system. The same investigator measured BP by auscultation 30 s before the end of each 2 min stage. Exercise stress test end points included any combination of the following: an overall rating of perceived exertion ≥ 18; no increase in oxygen uptake > 150 ml with increasing exercise intensity; a respiratory exchange ratio > 1.1; achievement of age predicted maximal HR; and/or inability to maintain pedaling cadence [25]. At the conclusion of the exercise test, subjects were attached to the same ambulatory monitor as after the orientation session to further acquaint them with the equipment.

Subjects completed three, 40 min experiments in random order on three separate days that were at least 48 hr apart to avoid acute exercise effects on BP [7,24]. Prior to the three experiments, a 20 gauge, 32 mm indwelling Teflon catheter was inserted into the antecubital vein of the right arm and kept patent with a 0.9% saline solution. Experiments were conducted at the same time of day for a given subject, and began with a 20 min baseline period. BP measurements taken during the baseline period were averaged, and this value was used as baseline BP for that experimental session. The three experiments consisted of two exercise bouts on a cycle ergometer at 40% VO_2_peak (LIGHT) and 60% VO_2_peak (MODERATE) for 30 min, and a 40 min non-exercise control session of seated rest. The 30 min exercise sessions were preceded by a 5 min warm up and followed by a 5 min cool down for a total 40 min of exercise. BP was measured by auscultation during the experiments and over the course of 9 hr after the experiments using the Accutracker II automatic non-invasive ambulatory BP monitor. The average attachment time was 12:30 pm and the monitor was worn until waking the next morning.

### Blood Sampling and Analysis

Fasting blood samples were drawn into EDTA vacutainer tubes for insulin, glucose and lipid-lipoproteins determinations. Blood samples were also obtained approximately 2 hr after the standard meal for glucose and insulin determinations at the end of the baseline period, at 30 min of each 40 min experiment, and at 15 and 45 min after the experimental sessions while subjects were still in the laboratory but prior to attachment to the ambulatory BP monitor. Samples were drawn into EDTA vacutainer tubes and centrifuged at 2500 g and 23°C for 6 min. Plasma concentrations of insulin were measured in duplicate by radioimmunoassay using a solid phase, single antibody assay (Coat-a-Count Insulin, TKIN2, Diagnostic Products Corporation, Los Angeles, CA) with an intra-assay coefficient of variation (CV) of 7.0% and inter-assay CV of 7.8%. A multi-level control (CON6 Multivalent Control Module, Diagnostic Procedures Corporation) was analyzed during each insulin test run. Plasma glucose concentrations were determined in duplicate by an automated glucose oxidase method (Yellow Springs Instruments, Model 2003, Yellow Springs, OH). The intra- and inter-assay CV for the plasma glucose determinations were 2.8% and 3.6%, respectively, at 4.7 mmol·L^-1^. The homeostasis model assessment (HOMA) of insulin resistance was calculated with the formula: [fasting glucose (mg·dL^-1^) × insulin (ulU·mL^-1^)]·22.5^-1 ^[26]. Fasting blood lipids and lipoproteins were determined by oxidase assays using colorimetric enzymatic methods (Cobras^® ^Integra™, Roche Diagnoxtics, Mannheim, Germany). The intra- and inter-assay CV for the triglyceride assays were 1.6% and 1.9%, respectively. Low density lipoprotein was calculated with the Friedewald equation [27].

### Sample Size Estimates

Sample size power calculations were conducted assuming a multivariate approach to analyzing repeated measure BP data [28]. Based upon our previous work [5,20,24], a series of power assessments were fit to estimated BP means and standard deviations for the experimental conditions and Msyn groups. This prior research indicated adequate power could be expected to detect differences in the BP change from baseline among the experimental conditions and Msyn groups with BP hourly change correlations of 0.75 across time. Based upon these assumptions, sample sizes of 30 men in each of the Msyn groups were sufficient to provide adequate power for detecting a SBP within method effect (non-exercise control, LIGHT and MODERATE) with a power of 1.000 and a SBP method by Msyn interaction between Msyn group effect with a power of 0.600.

### Statistical Analyses

Descriptive statistics were obtained on all study parameters. BP was averaged at hourly intervals over the course of 9 hr after the experimental sessions; the time period over which PEH was noted when the men were awake and ambulating [24]. Pearson correlations examined relationships among variables that may influence the BP response to exercise. These variables included age, baseline BP, body mass index, waist circumference, VO_2_max, HOMA, fasting insulin, glucose and lipids and lipoproteins, and the change in insulin and glucose during the experiments compared to baseline. Distributions were determined for these variables using the Kolmogorov-Smirnov test. The only variables that violated assumptions of normality were the change in insulin and glucose during the experiments compared to baseline. They were subsequently transformed into logarithms to improve the skewed distributions. Independent t-tests determined if there were differences in subject characteristics among the men with and without the metabolic syndrome.

**Figure 1:**
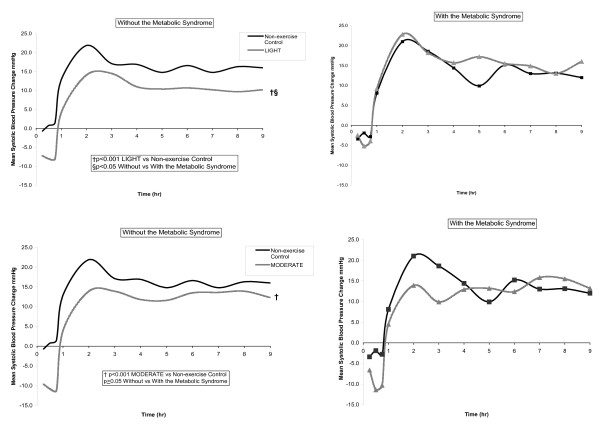
The average adjusted systolic blood pressure change from baseline after LIGHT and MODERATE compared to non-exercise control at hourly intervals over 9 hr among men without and with the Msyn.

**Figure 2:**
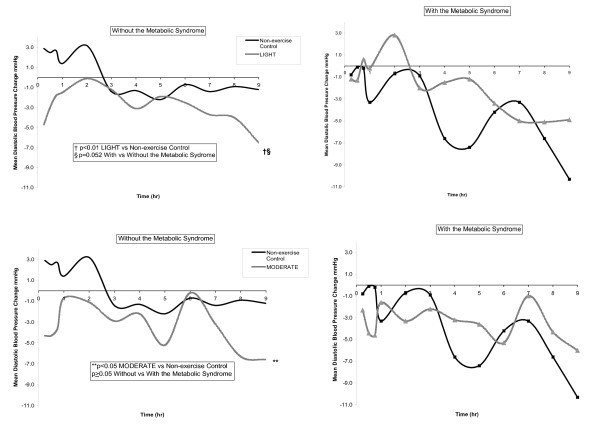
The average adjusted diastolic blood pressure change from baseline after LIGHT and MODERATE compared to non-exercise control at hourly intervals over 9 hr among men without and with the Msyn.

Three by two way repeated measures ANCOVA with HOMA as a covariate determined if BP, insulin and glucose differed over time among experimental conditions (non-exercise control, LIGHT, MODERATE) among men without and with the Msyn. Significant interaction effects were found between BP and men without the Msyn. Therefore, three way repeated measures ANCOVA then determined if BP, insulin and glucose differed over time among experimental conditions (non-exercise control, LIGHT, MODERATE) *within *the Msyn groups, and these results are displayed in Table 2. In order to determine *between *Msyn group differences, two by two way repeated measures ANCOVA determined if BP, insulin and glucose differed over time among exercise (LIGHT or MODERATE) and non-exercise control among men without and with the Msyn, and these results are displayed in Figures 1 and 2.

Multivariable regression analyses were performed to examine what factors correlated with the BP change from baseline following exercise compared to non-exercise control over 9 hr. All statistical analyses were done with the Statistical Package for Social Sciences Base 14.0 for Windows (SPSS Inc., Chicago, IL) with p < 0.05 established as the level of statistical significance.

## Results

### Subjects

Participants were 46 overweight, middle-aged Caucasian men with pre- to Stage 1 hypertension, borderline dyslipidemia [2], and low cardiovascular fitness [25] (Table 1). Of these, 39.1% had the Msyn [2]. Men with the Msyn had higher fasting insulin, HOMA, total cholesterol/high density lipoprotein-cholesterol ratio, and triglycerides and lower high density lipoprotein-cholesterol than men without the Msyn (Table 1) (p < 0.05).

**Table 1:** Mean (± SEM) fasting physical characteristics of the total sample and among those without and with the metabolic syndrome *

		Metabolic Syndrome	
		
Variable	Total Sample (n = 46)	Without (n = 28)	With (n = 18)
Age (yr)	44.3 ± 1.3	45.0 ± 1.8	43.2 ± 2.1
Maximum Oxygen Consumption (ml·kg^-1^·min^-1^)	30.7 ± 0.9	30.7 ± 1.1	30.6 ± 1.5
Body Mass Index (kg/m^2^)	29.5 ± 0.7	29.0 ± 1.1	30.4 ± 0.6
Waist Circumference (cm)	102.4 ± 2.1	100.7 ± 3.1	105.0 ± 2.5
Awake Systolic Blood Pressure (mmHg)**	145.5 ± 1.6	145.6 ± 2.2	145.4 ± 2.4
Awake Diastolic Blood Pressure (mmHg)	86.3 ± 1.2	86.9 ± 1.6	85.3 ± 1.3
**Insulin **(pmol·L^-1^)	80.0 ± 8.7	59.5 ± 7.0†	111.8 ± 17.1
Glucose(mmol·L^-1^)	4.5 ± 0.1	4.4 ± 0.1	4.6 ± 0.1
**Homeostasis Model Assessment**	2.3 ± 0.3	1.7 ± 0.2†	3.3 ± 0.6
Total Cholesterol (TCHOL) (mmol·L^-1^)	5.0 ± 0.2	4.9 ± 0.2	5.1 ± 0.3
**High Density Lipoprotein-Cholesterol **(mmol·L^-1^)	1.10 ± 0.03	1.21 ± 0.04‡	0.93 ± 0.03
Low Density Lipoprotein-Cholesterol (mmol·L^-1^)	3.1 ± 0.2	3.2 ± 0.2	3.1 ± 0.3
**Total Cholesterol/High Density Lipoprotein Ratio **(U)	4.7 ± 0.2	4.2 ± 0.2‡	5.4 ± 0.2
**Triglycerides **(mmol·L^-1^)	1.6 ± 0.2	1.1 ± 0.1‡	2.4 ± 0.3

* National Cholesterol Education metabolic syndrome diagnosis is established when three of five criteria are present [2]. The fasting criteria include: waist circumference > 102 cm; triglycerides ≥ 1.7 mmol·L^-1^; high density lipoprotein-cholesterol <1.2 mmol·L^-1^; blood pressure ≥ 130 and/or 85 mmHg; and glucose > 6.0 mmol·L^-1^

** Awake blood pressure= ambulatory blood pressure averaged over the time period when men were awake and ambulating, i.e., day time blood pressure

†p < 0.01, p < 0.001 ‡ without vs with the Metabolic Syndrome

### Blood Pressure Response

#### Within Msyn Group Effect

Table 2 displays the adjusted BP change from baseline after the experiments over 9 hr. Among men without the Msyn, SBP was 6.4 ± 1.9 mmHg lower from baseline after LIGHT and 6.0 ± 1.6 mmHg lower after MODERATE compared to non-exercise control (p < 0.001). Similarly, DBP was reduced 3.1 ± 1.5 mmHg from baseline after LIGHT (p < 0.01) and 2.4 ± 1.3 mmHg (p < 0.05) after MODERATE compared to non-exercise control among men without the Msyn. The post exercise BP response from baseline was not different after LIGHT and MODERATE versus non-exercise control among men with the Msyn (p ≥ 0.05).

**Table 2:** Within group adjusted blood pressure change from baseline after exercise and non-exercise control over 9 hr among the men without and with the metabolic syndrome {Mean ± SEM (95% Confidence Interval)}*

	SBP Change (mmHg)			DBP Change (mmHg)		
	
	Non-Exercise Control	LIGHT	MODERATE	Non-Exercise Control	LIGHT	MODERATE
Without Baseline (n = 28)	122.6 ± 3.3			86.4 ± 2.3		
Post-Pre Experiment Change	12.4 ± 1.7 (9.0,15.8)	**6.0 ± 1.7‡§ **(2.6,9.4)	**6.4 ± 1.6‡ **(3.0,9.7)	0.3 ± 1.2 (-2.1,2.7)	**-2.8 ± 1.0† **(-5.0,-0.7)	-2.1 ± 1.0** (-4.0,-0.2)
With Baseline (n = 28)	126.7 ± 2.6			85.7 ± 1.6		
Post-Pre Experiment Change	9.8 ± 2.1 (5.5,14.1)	10.9 ± 2.1 (6.6,15.2)	6.9 ± 2.1 (2.7,11.2)	-3.7 ± 1.5 (-6.8,-0.6)	-1.9 ± 1.4 (-4.6,0.9)	-3.5 ± 1.2(-6.0,-1.0)

* Baseline = Adjusted blood pressure (Mean ± SEM) of the pre-experiment 20 min period of seated rest for non-exercise control, LIGHT and MODERATE. Baseline BP did not differ among experiments so that average baseline BP for the three experiments is presented in Table 2; Post-Pre Experiment Change = Hourly blood pressure change after the experiment minus baseline blood pressure averaged over 9 hr.

**p < 0.05, †p < 0.01, ‡p < 0.001 exercise vs non-exercise control; §p < 0.05 exercise vs non-exercise control with vs without the metabolic syndrome.

LIGHT, 40% VO_2_peak; MODERATE, 60% VO_2_peak.

#### Between Msyn Group Effect

Figures 1 (SBP) and 2 (DBP) display the BP response from baseline at hourly intervals after exercise (LIGHT and MODERATE) compared to non-exercise control over 9 hr among men without and with the Msyn. SBP was 7.5 ± 3.2 mmHg lower from baseline after LIGHT compared to non-exercise control among men without the Msyn (-6.4 ± 1.9 mmHg) versus men with the Msyn (1.1 ± 2.4 mmHg) (Figure 1) (p < 0.05). Similarly, DBP tended to be 4.9 ± 2.5 mmHg lower from baseline after LIGHT compared to non-exercise control among men without the Msyn (-3.1 ± 1.5 mmHg) versus men with the Msyn (1.8 ± 1.9 mmHg) (Figure 2) (p = 0.052). The post exercise BP response from baseline was not different after MODERATE compared to non-exercise control between Msyn groups (p ≥ 0.05).

#### Insulin and Glucose Response

**Figure 3:**
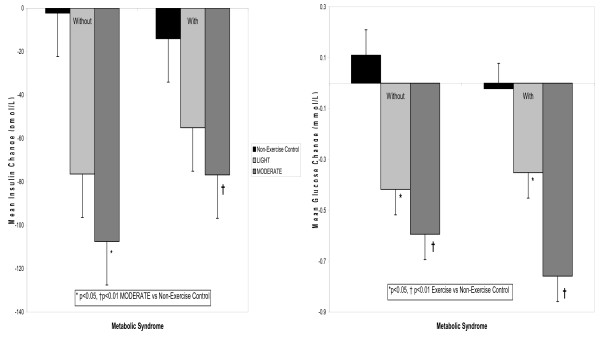
**The average insulin and glucose change from baseline during the experiments among men without and with the Msyn.** LIGHT = 40% VO_2_peak; MODERATE = 60% VO_2_peak.

Figure 3 displays the insulin and glucose response from baseline during the experiments. Insulin decreased from baseline during all experiments among the men without and with the Msyn (p < 0.01). The insulin decrease from baseline was greater during MODERATE (p < 0.05) but not during LIGHT (p ≥ 0.05) compared to non-exercise control among men without and with the Msyn. Glucose decreased from baseline after exercise compared to non-exercise control during all experiments among men without and with the Msyn (p < 0.05). However, there was no difference in the insulin and glucose responses from baseline during exercise compared to non-exercise control between Msyn groups (p ≥ 0.05).

#### Relationships among Blood Pressure and Components of the Cardiometabolic Profile

Correlates of the BP change from baseline after LIGHT versus non-exercise control are shown in Table 3. Factors accounting for 17% of the SBP response after LIGHT were baseline SBP (β = -0.351, r^2 ^= 0.123, p = 0.020), Msyn (β = 0.277, r^2 ^= 0.077, p = 0.069), and HOMA (β = -0.124, r^2 ^= 0.015, p = 0.424). Whereas, Msyn (r^2 ^= 0.096, p = 0.036) was the only significant correlate of the DBP response after LIGHT.

**Table 3:** Correlates of the average blood pressure change from baseline after exercise versus non-exercise control over 9 hr.

**Blood Pressure Change**	**Predictors**	**β**	**t**	**Partial r**	**r**^**2**^	**p**
**LIGHT Versus Non-Exercise Control**						

**Systolic Blood Pressure**	Baseline Systolic Blood Pressure	-0.233	-2.426	-0.351	0.123	**0.020**
	Metabolic Syndrome	5.807	1.869	0.277	0.077	0.069
	HOMA	-0.645	-0.807	-0.124	0.015	0.424
Model Summary				0.474	0.170	0.013
**Diastolic Blood Pressure**	Baseline Diastolic Blood Pressure	-0.045	-0.361	-0.056	0.003	0.720
	Metabolic Syndrome	4.852	1.918	0.284	0.081	0.062
	HOMA	-0.101	-0.154	-0.024	0.001	0.878
Model Summary				0.315	0.035	0.216

**MODERATE Versus Non-Exercise Control**						

**Systolic Blood Pressure**	Baseline Systolic Blood Pressure	-0.131	-1.428	-0.215	0.046	0.161
	Metabolic Syndrome	2.628	0.928	0.142	0.020	0.358
	HOMA	0.062	0.084	0.013	0.000	0.934
Model Summary				0.028	0.014	0.315
**Diastolic Blood Pressure**	Baseline Diastolic Blood Pressure	-0.055	-0.500	-0.077	0.006	0.620
	Metabolic Syndrome	2.571	1.111	0.169	0.029	0.273
	HOMA	0.502	0.829	0.127	0.016	0.412
Model Summary				0.288	0.018	0.300

HOMA = homeostasis model assessment

## Discussion

This study examined the influence of the Msyn on the immediate antihypertensive effects of aerobic exercise among men with pre- to Stage 1 hypertension after lower intensity, aerobic exercise. Among men without the Msyn, SBP was 8 mm Hg (Figure 1) and DBP 5 mm Hg (Figure 2) lower after LIGHT compared to non-exercise control, but not after MODERATE over 9 hr. Among men with the Msyn, BP was not different after exercise and non-exercise control. Contrary to our hypothesis, men without the Msyn responded more favorably to the immediate antihypertensive effects of lower intensity, aerobic exercise than men with the Msyn. The magnitude of the exercise-induced BP differences we found between Msyn groups has important clinical and public health considerations for the personalization of exercise prescriptions for those with hypertension, 40% of who have the Msyn [4].

The reasons that the men without the Msyn responded more favorably to the antihypertensive effects of LIGHT than men with the Msyn are unclear. The vasoactive properties of insulin are greater with increasing exercise intensity in order to augment the disposal of glucose into the skeletal muscles [29-31]. However, insulin's vasoactive role in skeletal muscle perfusion is paradoxical having both hypotensive, i.e., acutely decreasing vascular resistance and BP; and pro-hypertensive effects, i.e., activation of renal sodium reabsorption and the sympathetic nervous system that serve to elevate BP [13,18,32,33]. Our work [21-24] and that of others [34] supports the notion that the immediate BP lowering effects of aerobic exercise are dependent upon the vasodilator-vasoconstrictor balance imposed on the vasculature by interactions among genetic predispositions, exercise intensity, and the depressor and pressor actions of BP regulatory hormones such as catecholamines, renin and insulin.

The paradoxical vasoactive role of insulin on skeletal muscle perfusion would appear to offer a possible explanation for our finding that men without the Msyn responded to LIGHT as antihypertensive therapy; whereas the men with the Msyn did not. For these reasons, we explored in multivariable regression analyses factors that could account for the post exercise BP response. The insulin and glucose responses from baseline during exercise did not differ between the Msyn groups (Figure 3), offering no insight for our findings. Bursztyn et al. [35] assessed the relationships among insulin, glucose and ambulatory BP before and after 14 wk of aerobic exercise training performed 3 d/wk at 60–70% of peak HR among middle aged adults with hypertension. Despite a marked reduction in insulin and glucose, ambulatory BP was not different as a result of exercise training. Their findings are consistent with ours in that the exercise-induced insulin and glucose responses appear not to associate with the BP response to aerobic exercise.

Rheaume and co-investigators studied the effects of acute moderate intensity (50% VO_2_peak for 30 min) [36] and prolonged, vigorous intensity (70% VO_2_peak for 90 min) [37] aerobic exercise on insulin sensitivity as assessed by an intravenous glucose tolerance test among men with elevated BP for up to 4 hr after exercise. They found that insulin sensitivity was increased after moderate but not vigorous intensity exercise. Rheaume et al. [36,37] partially attributed these differential exercise intensity to a higher adrenergic response and greater vasoconstrictor actions after vigorous than moderate intensity exercise.

The results of Rheaume et al. [36,37] provide insight into why men without the Msyn but not men with the Msyn lowered BP following exercise in our study. As shown in Table 1, men without the Msyn had increased insulin sensitivity (i.e., lower HOMA) compared to men with the Msyn (Table 1). Among men without the Msyn, as insulin sensitivity increased, the decreases in SBP from baseline after LIGHT (r = 0.403, r^2 ^= .162, p = 0.019) and MODERATE (r = 0.440, r^2 ^= 0.193, p = 0.033) were greater. Furthermore, in multivariable regression analyses, HOMA remained a significant correlate of the SBP response following LIGHT (Table 3). In contrast, among men with the Msyn, as insulin sensitivity decreased, the decrease in SBP from baseline after LIGHT (r = -0.464, r^2 ^= 0.215, p = 0.053) tended to be greater but not after MODERATE (r = -0.277, r^2 ^= 0.077, p = 0.266). The antihypertensive benefits of lower intensity, aerobic exercise appeared to result from improved insulin sensitivity and a more favorable vasodilator-vasoconstrictor balance following exercise among men without the Msyn compared to men with the Msyn. Future investigation is needed to more precisely establish the mechanisms for our observations.

This study has several limitations. Post-prandial plasma insulin and glucose assessments were measured only in the laboratory and not under ambulatory conditions in an attempt to minimize experimental intrusions that could affect the BP measurements and for reasons of subject convenience. However, we examined the relationships among BP, insulin and glucose in the laboratory (data not shown) and found the findings to be strikingly similar to those reported in this paper over 9 hr. We did not perform an oral glucose tolerance test to determine insulin sensitivity during and after exercise while subjects were still in the laboratory. It remains possible that a more sensitive measure of insulin sensitivity than HOMA may have shown different associations with post exercise BP. Lastly, we attempted to control for the confounding influence of diet on the baseline plasma insulin and glucose concentrations of our subjects prior to the experiments by instructing them to consume a standard meal 2 hr prior to any testing. However, we relied upon self-report to verify dietary compliance which does not ensure the instructions were followed.

## Conclusion

Men without the Msyn appear to respond more favorably to the immediate antihypertensive effects of lower intensity, aerobic exercise than men with the Msyn. Factors accounting for this result were baseline BP, the Msyn and HOMA, explaining 17% of the variability in the SBP response after LIGHT. Future work is needed to confirm our findings in a larger, more ethnically diverse sample of men and women to elucidate reasons for the differential impact that the Msyn has on the antihypertensive effects of aerobic exercise. Considering the significant public health burden of hypertension and the Msyn and the importance of exercise in their prevention, treatment and control, results from such studies could yield important new knowledge for the personalization of exercise prescriptions among those with hypertension and the Msyn.

## Authors' contributions

LSP: Concept and design; data acquisition; statistical expertise; data analysis and interpretation; and primary manuscript writer. BEB: Data acquisition; statistical expertise; data analysis and interpretation; and manuscript reviewer. JLVH: Data acquisition; data interpretation; and manuscript reviewer for important intellectual content. CMM: Data acquisition; data interpretation; and manuscript reviewer for important intellectual content. HG–D: statistical expertise; data analysis and interpretation; and manuscript reviewer for important intellectual content. PDT: Data acquisition; data interpretation; and manuscript reviewer for important intellectual content. All authors have read and approved the final manuscript.

## Pre-publication history

The pre-publication history for this paper can be accessed here:


